# The association of previous night's sleep duration with cognitive function among older adults: a pooled analysis of three Finnish cohorts

**DOI:** 10.1007/s10433-023-00779-6

**Published:** 2023-08-03

**Authors:** Tea Teräs, Saana Myllyntausta, Marika Salminen, Laura Viikari, Katja Pahkala, Olli Muranen, Nina Hutri-Kähönen, Olli Raitakari, Suvi Rovio, Sari Stenholm

**Affiliations:** 1 Department of Public Health, University of Turku and Turku University Hospital, Turku, Finland; 2 Centre for Population Health Research, University of Turku and Turku University Hospital, Turku, Finland; 3 Department of Psychology and Speech-Language Pathology, University of Turku, Turku, Finland; 4 Welfare Division, City of Turku, Turku, Finland; 5 Department of General Practice, Faculty of Medicine, University of Turku and Turku University Hospital, Turku, Finland; 6 Department of Geriatric Medicine, Faculty of Medicine, University of Turku, Turku City Hospital, Turku, Finland; 7 Research Center of Applied and Preventive Cardiovascular Medicine, University of Turku, Turku, Finland; 8 Paavo Nurmi Centre & Unit for Health and Physical Activity, University of Turku, Turku, Finland; 9 Department of Pediatrics, Tampere University Hospital and Faculty of Medicine and Health Technology, Tampere University, Tampere, Finland; 10 Department of Clinical Physiology and Nuclear Medicine, Turku University Hospital, Turku, Finland; 11 Research Services, Turku University Hospital and University of Turku, Turku, Finland

**Keywords:** Sleep quantity, Sleep duration, Cognitive function, CANTAB, Accelerometry

## Abstract

**Study objectives:**

Sleep duration has been shown to associate with cognitive function, but little is known about the short-term effect of sleep duration on the previous night. This study examines how usual sleep duration and previous night’s sleep duration are associated with cognitive function in older adults.

**Methods:**

The study population consisted of 2949 adults aged 59–92 years (mean 72.6, SD 5.7) derived from three Finnish cohorts. Participants’ self-reported usual sleep duration was categorized into short (< 7 h, 19%), mid-range (7– < 9 h, 64%), and long (≥ 9 h, 17%). Self-reported sleep duration on the night prior to cognitive testing was categorized into shorter (59%), same (35%), and longer (5.9%) than usual sleep duration. Computerized Cambridge Neuropsychological Test Automated Battery (CANTAB®) was used to assess: (1) learning and memory, (2) working memory, (3) information processing, and (4) reaction time.

**Results:**

Participants with self-reported long, but not short, usual sleep duration had poorer learning and memory (*p* = .004), information processing (*p* = .003), and reaction time (*p* = .006) when compared to those with mid-range sleep duration. Those who slept more than usually the night prior to cognitive testing had poorer information processing (*p* = .019) than those sleeping the same as usually, while sleeping less than usually was not associated with cognitive function.

**Conclusions:**

This study suggests that while long sleep duration was associated with worse cognitive function, sleeping more than usually the night prior to cognitive testing was only associated with information processing, and sleeping less than usually is not associated with cognitive function.

## Introduction

Sleep duration has been previously linked to cognitive function with both short and long sleepers having poorer cognitive function than mid-range sleepers. This inverted U-shaped association has been widely shown in studies focusing on overall cognition (Ding et al. 2020; Gildner et al. 2014; Ma et al. 2020; Zhang et al. 2021), but the specific cognitive domains affected are still unclear. Previous studies on sleep duration and specific cognitive domains have mainly focused on different aspects of memory (Kondo et al. 2021; Okuda et al. 2021; Xu et al. 2014, 2011), while other cognitive domains have been scarcely examined (Blackwell et al. 2011).

Several health behaviors prior to cognitive testing, such as recent physical activity (Clark et al. 2022; Sng et al. 2018), caffeine intake (Einöther and Giesbrecht 2013), and alcohol hangover (Devenney et al. 2019), are shown to affect test performance. However, it remains unclear how small short-term changes in sleep duration, such as a 1-h change from usual sleep duration for one night, affect the performance in cognitive testing. This has practical relevance as patients and study participants often complain about being anxious and consequently sleeping poorly the night prior to clinical examinations.

Most of the previous research on sleep during the night prior to cognitive testing has been conducted in laboratory settings with small and selected study populations. One night of total sleep deprivation has been shown to alter brain connectivity (Pesoli et al. 2022) and to affect cognitive function similarly to alcohol intoxication (Dawson and Reid 1997). Additionally, a meta-analysis of short-term total sleep deprivation among healthy adults showed that a total sleep deprivation period of 24–48 h significantly reduces function in several cognitive domains, such as complex attention and working memory (Lim and Dinges 2010). The few non-laboratory studies have examined the effect of 5–7 nights of restricted or extended sleep duration on cognitive function and found that a partial sleep deprivation for 5 days impaired working memory (del Angel et al. 2015), while a sleep extension of 7 days had a beneficial effect on daytime alertness and reaction time (Kamdar et al. 2004). However, a recent study found no association between 6 days of sleep extension and sustained attention, spatial rotation ability, mental flexibility, or working memory (Clark et al. 2022).

The few studies focusing primarily on previous night’s sleep among free-living adults have shown mixed results. Some studies have found an association between previous night’s sleep duration and various cognitive domains, such as alertness (Kalanadhabhatta et al. 2021; Neylan et al. 2010), working memory (O’Brien et al. 2012), and self-reported daytime functioning (Smith et al. 2015) (i.e., questionnaire based on Daytime Insomnia Symptom Scale (Buysse et al. 2007)). On the other hand, previous night’s sleep duration has not been found to be associated with global cognition (Seelye et al. 2015) or calculation performance (Kalanadhabhatta et al. 2021). However, only some of these previous studies have obtained sleep duration with objective measures, such as in-home sensors (Seelye et al. 2015), commercial fitness trackers (Kalanadhabhatta et al. 2021), and actigraphy (Neylan et al. 2010). Additionally, it remains obscure how sleeping more or less than usual the night prior to cognitive testing affects the cognitive function, as previous studies have not taken into account participants’ usual sleep duration when examining the previous night’s sleep duration.

The aim of the current study was to evaluate how usual sleep duration is associated with performance in cognitive tests measuring several different cognitive domains. An additional aim was to examine how sleeping less, the same, or more than usually the night prior to cognitive testing is associated with different cognitive domains. To elucidate the role of usual and previous night’s sleep duration on cognitive function among older adults, we examined how self-reported usual sleep duration is associated with performance in several cognitive domains by leveraging the data from three Finnish cohorts. Additionally, we examined how sleep duration during previous night in comparison with usual sleep duration is associated with performance in several cognitive domains. Finally, we conducted the analyses utilizing data on accelerometer-measured sleep duration that has been collected in one of the cohorts.

## Methods

### Study cohorts and participants

The study population consisted of participants from three Finnish cohorts: the Finnish Retirement and Aging Study (FIREA), the Cardiovascular Risk in Young Finns Study (YFS), and the Turku Senior Health Clinic Study (TSHeC).

The FIREA study is an ongoing longitudinal cohort study of older public sector workers in Finland established in 2013. Detailed description of the FIREA study design has been reported elsewhere (Leskinen et al. 2018). Finnish-speaking participants with an estimated retirement date between 2017 and 2019, who lived in Southwest Finland and were still working, were invited to participate in the clinical sub-study (n = 773). Of them, 290 (38%) participated in the sub-study between September 2015 and May 2018 (Teräs et al. 2020).

The YFS is a long-standing national multi-center study originally designed to provide evidence on the importance and timing of early life exposures in the development of cardiovascular diseases (Raitakari et al. 2008). The first examination in 1980 recruited 3596 participants aged 3–18 years from five cities and their surrounding rural communities. The cohort has been followed up regularly every 3–6 years. In the most recent follow-up study in 2018–2020, the data collections were expanded to include information not only from the original participants, but also from the parents and offspring of the original participants. For this study, we used data collected from the parents of the original YFS participants. In total, 3940 persons were invited to participate in a detailed clinical examination. Of those, 2149 came to clinical visit and 2055 underwent cognitive testing (aged 59–93 years).

The TSHeC population consists of all home-dwelling citizens of city of Turku, who were born in 1945, in the beginning of 2020 (n = 2044). Those with municipal home care (n = 196) were excluded, 33 were deceased before the invitation, 391 refused to participate, and 128 were not reached, leaving 1296 subjects for the senior health clinic study sample. All participants who came to a clinic visit between January 2020 and February 2021 were invited to participate in a detailed cognitive testing (n = 935), and of those, 766 (82%) participated.

**Fig. 1:**
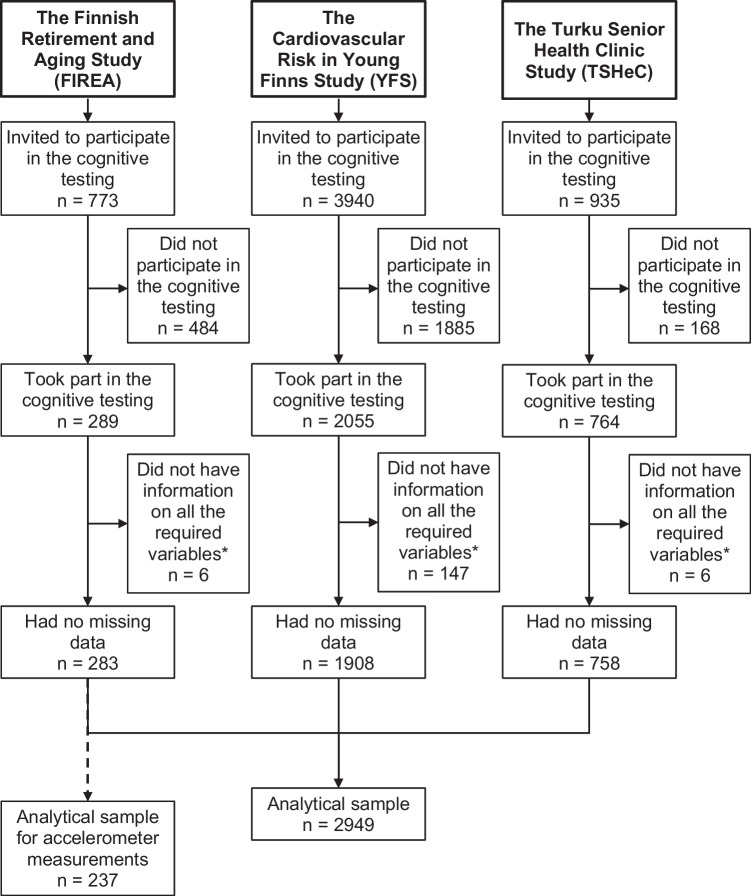
Flow chart for the analytical sample of the study. *Note.* *These included self-reported usual sleep duration, self-reported sleep duration on the night prior to cognitive testing, at least one of the examined cognitive domains, or the covariates (age, sex, socioeconomic status, and season of the measurements)

To be included in this study, the participants from each cohort needed to have data on self-reported usual sleep duration, self-reported sleep duration on the night prior to cognitive testing, at least one of the examined cognitive domains, and the applied covariates (age, sex, socioeconomic status, and season of the measurements). This resulted in an analytical sample of 2949 persons (the FIREA: *n* = 283; the YFS *n* = 1908; and the TSHeC: *n* = 758). The selection of the analytical sample is illustrated in Fig. 1. Informed consent was obtained from all participants. Each study was conducted in accordance with the Helsinki declaration and was approved by local ethics committees.

### Assessment of sleep

In the FIREA study, information on usual sleep duration was based on participants’ responses to the survey question “How many hours do you usually sleep per 24 h?”. Participants answered by choosing one of the following response alternatives: 6 h or less, 6.5, 7, 7.5, 8, 8.5, 9, 9.5, and 10 h or more. In the YFS, the usual sleep duration was queried separately for weekdays and weekend days with the following response categories: 1, 2, 3, 4, 4.5, 5, 5.5, 6, 6.5, 7, 7.5, 8, 8.5, 9, 9.5, 10, 10.5, 11, 11.5, 12, 13, and 14 h. Participants’ usual sleep duration was calculated using the formula: (5 * usual sleep duration on weekdays + 2 * usual sleep duration on weekends) / 7. In the TSHeC, participants were queried “What time do you usually go to sleep?” and “What time do you usually wake up in the morning?”. Based on these questions, usual sleep duration was calculated at 0.5-h intervals. For the analyses, self-reported usual sleep duration was categorized into three groups: short (< 7 h), mid-range (7 h to < 9 h), and long (≥ 9 h) sleep duration based on recommendations by the National Sleep Foundation on the optimal duration of sleep (Hirshkowitz et al. 2015).

Self-reported sleep duration on the night preceding the cognitive testing was assessed similarly in all three cohorts. During the clinical visit before taking part in the cognitive testing, the participants were asked to report their sleep duration on the previous night. Based on the usual sleep duration and the sleep duration on the night prior to cognitive testing, we formed a variable to indicate the difference between the self-reported sleep duration on the night before cognitive testing and the self-reported usual sleep duration. The participants were categorized into three groups based on this difference: shorter than the usual sleep duration (i.e., those who slept at least an hour less on the night preceding the cognitive testing than usually), same as the usual sleep duration (i.e., those who slept less than an hour more or less on the night preceding the cognitive testing than usually), and longer than the usual sleep duration (i.e., those who slept at least an hour more on the night preceding the cognitive testing than usually).

In addition, accelerometer-measured sleep was available in the FIREA cohort. The wrist-worn triaxial wActiSleep-BT accelerometer by ActiGraph (Pensacola, Florida, US) was initialized to record movements during sleep and wakefulness at 80 Hz. The participants received the device via mail before the cognitive testing and were instructed to wear it continuously on their non-dominant wrist for 24 h per day for at least seven days and nights (including at least two workdays and two free days). The manufacturer’s ActiLife software (ActiGraph, Pensacola, Florida, USA) was used to download and convert the raw data into 60-s epochs. We used the Cole-Kripke algorithm (Cole et al. 1992) in defining all the epochs as either sleep or wake and the ActiGraph algorithm (Actigraph 2018) in detecting sleep periods. The data-handling and checking procedures as well as the used algorithms have been described in more detail elsewhere (Myllyntausta et al. 2020). From the accelerometer data, we obtained information on sleep duration on the night preceding the cognitive testing as well as the average sleep duration of the whole measurement period (excluding the night preceding the cognitive testing) to indicate participants’ usual sleep duration. To be included in the accelerometer-based sub-analysis, the participants were required to have information on 1) their average sleep duration and sleep duration on the night before cognitive testing based on the accelerometer measurements, 2) data from at least one of the examined cognitive tests, and 3) information about the applied covariates (age, sex, occupational position, and season of the measurements). This resulted in an analytic sample of 237 participants from the accelerometer measurements. Accelerometer-based usual sleep duration was categorized similarly as self-reported into short (< 7 h), mid-range (7 h to < 9 h), and long (≥ 9 h), and the difference between usual sleep duration and sleep duration the night prior to cognitive testing into 1) shorter than the usual sleep duration, 2) same as the usual sleep duration, and 3) longer than the usual sleep duration.

### Assessment of cognitive function

Cognitive function was measured in each cohort using the Cambridge Neuropsychological Test Automated Battery (CANTAB®), a computerized test battery covering multiple cognitive domains including learning and memory, working memory, information processing, and reaction time. CANTAB® is a widely used standardized computer-based method for assessing cognitive function (Rovio et al. 2016; Waller et al. 2016; Zitser et al. 2020). The four tests used in this study were: Paired Associates Learning (PAL) for visual *memory and learning,* Spatial Working Memory (SWM) for *working memory*, Rapid Visual Information Processing (RVP) for *information processing*, and Reaction Time (RTI) for *reaction time*. Each CANTAB® test produces several outcome variables, which were categorized using Z scores to reduce the number of variables and to gain components that would explain most of the variation in the data set. More specifically, each individual variable was transformed within each cohort into a scale with a mean of 0 and a standard deviation (SD) of 1. Average scores of all test-specific variables were calculated to represent the testwise score, that is the 4 cognitive variables used in the study. Finally, the variables were converted so that a higher value reflects better cognitive function.

### Assessment of covariates

All analyses were adjusted for age, sex, socioeconomic status, and the season during which the cognitive testing was conducted. For the participants of the FIREA cohort, information on their date of birth, sex, and occupational title was obtained from the pension insurance institute for the public sector (Keva Public Sector Pensions). For the YFS participants, information on their date of birth was obtained from the digital and population data services agency, and information about sex and level of education was derived from questionnaires. For the TSHeC participants, information on age, sex, and level of education was obtained from postal questionnaires. For all the participants, the date of the cognitive testing was used to derive the season of the measurement (i.e., spring, summer, autumn, or winter).

Participants’ socioeconomic status was defined based on either their occupational status (in the FIREA cohort) or their level of education (in the YFS and the TSHeC cohorts) and categorized into three groups: high (FIREA: upper grade non-manual occupation; the YFS and the TSHeC: higher education), intermediate (FIREA: lower grade non-manual occupation; the YFS and the TSHeC: intermediate education), or low (FIREA: service and manual occupation; the YFS and the TSHeC: basic education).

### Statistical analyses

Characteristics of the participants are presented as mean and SD for age and as frequencies and percentages for the categorical variables. The characteristics are reported for the study cohorts combined and separately. Characteristics of the study cohorts were compared using the Kruskal–Wallis test or the Chi-square test.

For the analyses, data from the three study cohorts were pooled. First, we used linear regression analyses to compare different cognitive functions (i.e., learning and memory, working memory, information processing, and reaction time) in groups categorized by self-reported usual sleep duration using mid-range sleep duration (7 h to < 9 h) as the reference group. The analyses were adjusted for age, sex, socioeconomic status, study cohort, and the season of the measurement. The results are reported as mean estimates and their 95% confidence intervals.

Second, we examined different cognitive functions by categories based on differences in self-reported sleep duration on the night before cognitive testing and the self-reported usual sleep duration (adjusted for age, sex, socioeconomic status, study cohort, usual sleep duration, and the season of the measurement). These results are reported as mean estimates and their 95% confidence intervals. Sleeping the same amount as usual was used as the reference group.

Finally, we examined differences in various cognitive domains between categories based on differences in accelerometer-measured sleep duration on the night before cognitive testing and the accelerometer-based usual sleep duration from the FIREA study (adjusted for age, sex, socioeconomic status, study cohort, season of the measurement, and usual sleep duration). Sleeping the same amount as usual was used as the reference group, and the results are reported as mean estimates and their 95% confidence intervals.

All statistical analyses were conducted using the SAS Statistical Package version 9.4 (SAS Institute).

## Results

Characteristics of the whole study population and the three separate cohorts are shown in Table 1. The mean age was 72.6 years (SD 5.7, range 59.0 to 92.2) in the whole study population, and on average the FIREA cohort was the youngest and the TSHeC was the oldest cohort (*p* < 0.0001). The majority of the study population were women (64%) and the sex distribution of the study cohorts varied; the FIREA study cohort consisted of a higher proportion of women (83%) than the other study cohorts (61–62%) (*p* < 0.0001). Differences between the cohorts were also observed in the distribution of the socioeconomic status groups (*p* < 0.0001) so that the proportion of people with intermediate socioeconomic status was larger in YFS and smaller in TSHeC, and the proportion of people with low socioeconomic status was smaller in the YFS and larger in the TSHeC than in whole study population. The season of the measurement also differed between the cohorts (*p* < 0.0001) with the highest proportion of measurement conducted during winter in the FIREA cohort, during spring in the YFS, and during autumn in the TSHeC. Additionally, the proportion of those with short sleep duration (< 7 h) was lower and the proportion of those with long sleep duration (≥ 9 h) was markedly higher in the TSHeC than in the other study cohorts (*p* < 0.0001).

**Table 1:** Characteristics of the whole study population and the individual study cohorts

	All		Finnish Retirement and Aging study		Cardiovascular Risk in Young Finns Study		Turku Senior Health Clinic Study		
	(n = 2949)		(n = 283)		(n = 1908)		(n = 758)		
	M	SD	M	SD	M	SD	M	SD	p-value
Age	72.6	5.7	62.4	1.0	73.1	5.6	75.0	0.0	< .0001
	n	%	n	%	n	%	N	%	
Sex									< .0001
Men	1074	36	49	17	736	39	289	38	
Women	1875	64	234	83	1172	61	469	62	
Socioeconomic status									< .0001
High	1008	34	100	35	650	34	258	34	
Intermediate	1162	39	98	35	879	46	185	24	
Low	779	26	85	30	379	20	315	42	
Season of measurement									< .0001
Spring	981	33	81	29	713	37	187	25	
Summer	517	18	28	10	391	20	98	13	
Autumn	832	28	70	25	497	26	265	35	
Winter	611	21	103	37	307	16	201	27	
Self-reported usual sleep duration									< .0001
Short (< 7 h)	574	19	73	26	452	24	49	6	
Mid-range (7 h – < 9 h)	1878	64	203	72	1234	65	441	58	
Long (≥ 9 h)	497	17	7	2	222	12	268	35	

Socioeconomic status: occupational status (in the FIREA cohort) or level of education (in the YFS and the TSHeC cohorts)

Cognitive function by self-reported usual sleep duration is shown in Table 2. Those with a long sleep duration had poorer learning and memory (*p* = 0.004), information processing (*p* = 0.003), and reaction time (*p* = 0.006) when compared to those with mid-range sleep duration. No differences were observed between short and mid-range sleepers in any cognitive functions. Cognitive functions by categories of self-reported usual sleep duration within each study cohort are shown in supplementary materials (Table S1).

**Table 2:** Association between self-reported usual sleep duration and cognitive function

Cognitive function	Self-reported usual sleep duration									Short vs mid-range, p-value	Long vs mid-range, p-value
	Short (< 7 h) (n = 574)			Mid-range (7 h– < 9 h) (n = 1878)			Long (≥ 9 h) (n = 497)				
	Mean estimate	95% CI		Mean estimate	95% CI		Mean estimate	95% CI			
Learning and memory^a^	− 0.02	− 0.08	0.04	− 0.01	− 0.05	0.02	− 0.10	− 0.17	− 0.04	.831	.004
Working memory^b^	0.01	− 0.05	0.07	− 0.002	− 0.04	0.04	0.03	− 0.04	0.09	.648	.412
Information processing^c^	− 0.01	− 0.08	0.06	− 0.03	− 0.07	0.02	− 0.15	− 0.22	− 0.07	.697	.003
Reaction time^d^	− 0.02	− 0.08	0.04	− 0.04	− 0.08	0.005	− 0.13	− 0.20	− 0.07	.654	.006

Adjusted for age, sex, socioeconomic status, study cohort, and the season of the measurement

The numbers are mean estimates and their 95% confidence intervals of z-score-based cognitive test scores

Within each cohort, cognitive test data were normalized with M = 0 and SD = 1. Higher value reflects better cognitive function

^a^Measured with paired associated learning test (CANTAB®)

^b^Measured with spatial working memory test (CANTAB®)

^c^Measured with visual information processing test (CANTAB®)

^d^Measured with reaction time test (CANTAB®)

Table 3 shows cognitive functions among groups with shorter than the usual sleep duration, same as the usual sleep duration and longer than the usual sleep duration during the night prior to cognitive testing. Those who slept more than usually had poorer information processing when compared to those who slept the same amount as usually (*p* = 0.019). There were no marked differences in cognitive function between shorter than the usual sleep duration and same as the usual sleep duration. The cohort-specific results are shown in supplementary materials (Additional file 1: Table S2).

**Table 3:** Association between self-reported previous night’s sleep duration compared to usual sleep duration and cognitive function

	Slept less than usual (n = 1734)			Slept the same amount (n = 1042)			Slept more than usual (n = 173)				
Cognitive function	Mean estimate	95% CI		Mean estimate	95% CI		Mean estimate	95% CI		Less vs. same, p-value	More vs. same, p-value
Learning and memory^a^	− 0.04	− 0.08	0.004	− 0.05	− 0.10	− 0.002	− 0.08	− 0.18	0.02	.652	.532
Working memory^b^	0.005	− 0.04	0.05	0.02	− 0.03	0.07	0.02	− 0.08	0.12	.491	.962
Information processing^c^	− 0.07	− 0.12	− 0.02	− 0.03	− 0.09	0.03	− 0.18	− 0.30	− 0.06	.211	.019
Reaction time^d^	− 0.07	− 0.11	− 0.02	− 0.05	− 0.11	0.002	− 0.10	− 0.21	0.004	.592	.358

Adjusted for age, sex, socioeconomic status, study cohort, usual sleep duration, and the season of the measurement

The numbers are mean estimates and their 95% confidence intervals of z-score-based cognitive test scores

Within each cohort, cognitive test data were normalized with M = 0 and SD = 1. Higher value reflects better cognitive function

^a^Measured with paired associated learning test (CANTAB®)

^b^Measured with spatial working memory test (CANTAB®)

^c^Measured with visual information processing test (CANTAB®)

^d^Measured with reaction time test (CANTAB®)

We also examined differences in the studied cognitive domains based on differences in accelerometer-measured sleep duration on the night before cognitive testing and the accelerometer-based usual sleep duration from the FIREA study (Table 4). The only difference that was observed was the poorer information processing among those who slept less than usually when compared to those who slept the same amount as usually (p = 0.030).

**Table 4:** Association between accelerometer-based previous night’s sleep duration compared to usual sleep duration and cognitive function

	Slept less than usual (n = 109)			Slept the same amount (n = 90)			Slept more than usual (n = 38)			Less vs. same, p-value	More vs. same, p-value
Cognitive function	Mean estimate	95% CI		Mean estimate	95% CI		Mean estimate	95% CI			
Learning and memory^a^	0.06	− 0.37	0.50	0.06	− 0.39	0.51	0.16	− 0.31	0.63	.965	.429
Working memory^b^	− 0.03	− 0.43	0.36	0.04	− 0.37	0.45	− 0.01	− 0.43	0.42	.393	.695
Information processing^c^	0.33	− 0.10	0.77	0.53	0.08	0.99	0.53	0.05	1.00	.030	.961
Reaction time^d^	− 0.03	− 0.37	0.31	0.09	− 0.27	0.44	− 0.06	− 0.43	0.30	.105	.134

Adjusted for age, sex, socioeconomic status, study cohort, usual sleep duration, and the season of the measurement

The numbers are mean estimates and their 95% confidence intervals of z-score-based cognitive test scores

Cognitive test data were normalized with M = 0 and SD = 1. Higher value reflects better cognitive function

^a^Measured with paired associated learning test (CANTAB®)

^b^Measured with spatial working memory test (CANTAB®)

^c^Measured with visual information processing test (CANTAB®)

^d^Measured with reaction time test (CANTAB®)

## Discussion

In this study of 2949 older adults from three Finnish cohorts, we found that when self-reported usual sleep duration was considered, participants with a long sleep duration showed poorer performance in learning and memory, information processing, and reaction time than those with mid-range sleep duration. On the other hand, when self-reported previous night’s sleep duration of the participants was compared to usual sleep duration, sleeping longer than usually was associated with poorer information processing when compared to sleeping the same amount as usually. No differences in cognitive function were observed between those who had shorter or the same sleep duration than usually.

The found associations between long sleep duration and different cognitive domains are in line with previous research (Basta et al. 2019; Chiu et al. 2016; Faubel et al. 2009; Low et al. 2019; Ramos et al. 2020, 2013; Schmutte et al. 2007; van Oostrom et al. 2018). This study contributes to the literature by examining associations with several cognitive domains using computer-based cognitive tests, which enabled us to study the association between sleep duration and specific cognitive domains instead of global cognitive function. A novel finding of this study is that long sleep duration is associated with poorer performance in information processing and reaction time. Waller et al. found that sleep duration was not, but sleep latency was, associated with information processing and reaction time in a smaller study sample of men in their late 50 s (Waller et al. 2016). Others have also found an association, although inconsistent, between sleep difficulties, but not sleep duration, and reaction time among late middle-aged people (Kyle et al. 2017). We found long sleep duration to be associated with learning and memory, which is in line with previous research (Kondo et al. 2021; Xu et al. 2014, 2011). We found no association between short or long sleep duration and working memory, and although one previous study did find long sleep duration to be associated with working memory (Okuda et al. 2021), others did not either (Seelye et al. 2015; Spira et al. 2017; Swanson et al. 2021). We did not find short sleep duration to be associated with any of the cognitive domains, while some have found short sleep duration to be associated with global cognition (Ding et al. 2020; Gildner et al. 2014; Zhang et al. 2021). In the current study, the National Sleep Foundation’s recommendations were used to categorize sleep duration groups (i.e., < 7 h for short sleep duration), while in most previous studies, a lower threshold for short sleep duration has been used (i.e., < 6 h(Ding et al. 2020; Gildner et al. 2014; Zhang et al. 2021) or ≤ 4 h(Ma et al. 2020)). However, our aim was not to examine the association between sleep deprivation and cognitive function but rather the sleep of a healthy aging population.

This study indicated that sleeping more than usually on the night prior to cognitive testing was associated with poorer information processing, while sleeping less than usually was not associated with any of the measured cognitive domains. The current results on the role of previous night's sleep duration on cognition provide further understanding on the topic, as previous literature is scarce, especially regarding short-term changes from one’s usual sleep duration. In our experience, having slept poorly during the previous night is a common comment among patients/study participants during clinical visits and tests, and many seem to believe that it has a large effect, for example, on the performance in cognitive testing. Indeed, a few non-laboratory studies among adults have shown that previous night’s short sleep duration is associated with worse performance in various cognitive domains (Neylan et al. 2010; O’Brien et al. 2012; Smith et al. 2015). However, there are only a few studies that have examined the previous night’s sleep characteristics in comparison with one’s usual sleep characteristics and their association with cognitive function, and these prior reports have primarily focused on sleep difficulties. However, it has been previously shown that long sleep duration is a result of residual confounding rather than the cause for cognitive decline. Thus, it is possible that also the association found in this study between sleeping more than usual the night prior to cognitive testing and cognitive function might be explained by residual confounding, such as insufficient sleep the days before cognitive testing. Additionally, there might be some unmeasured factors mediating the found association.

In this study, one’s usual sleep duration was taken into account by adjusting the analyses with it, as it has been previously suggested that the usual sleep characteristics affect the association between previous night’s sleep and cognitive function (Hennecke et al. 2021; Neylan et al. 2010; Yu et al. 2016). One study showed that longer previous night’s total sleep time was associated with better daytime functioning in both usually poor and good sleepers, while poorer sleep efficiency and greater wake after sleep onset were associated with poorer daytime functioning only among those who usually were poor sleepers (Smith et al. 2015). Similarly, Yu et al. showed in adolescents that among usually poor sleepers, previous night’s better-than-usual sleep was associated with better executive function, and, in contrast, among usually good sleepers, previous night’s better-than-usual sleep was associated with worse executive function (Yu et al. 2022). Additionally, a recent study examining chronic and acute sleep deficits showed that chronic sleep deprivation is associated with worse spatial working memory even after one night of recovery sleep (Hennecke et al. 2021).

Our findings suggest that cognitive test results can be regarded reliable despite patient’s complaints about previous night’s uncharacteristically short sleep duration. However, it has been shown that beliefs about the negative effects of poor sleep associate more strongly with impaired daytime functioning among those suffering insomnia compared to good sleepers (Smith et al. 2015). More research is needed to validate these results and to alter persons’ own beliefs on the topic.

To overcome the limitations regarding self-reported sleep duration, we conducted additional analyses in one of the cohorts, in which accelerometer data on sleep duration were available. While sleeping more than usually was associated with poorer performance in information processing when assessed with self-reports, in the case of accelerometer-based sleep duration, shorter sleep duration than usually was associated with poorer performance in information processing when compared to usual sleep duration. However, the accelerometer measurements were available only for a small female-dominated population in an occupational cohort limiting the generalizability of the results. It has also been previously shown that self-reports and accelerometry-based reports are only moderately correlated, with self-reports typically indicating longer sleep durations (Lauderdale et al. 2008; van den BERG et al. 2008). We have previously examined sleep duration measured both with accelerometer over one week and self-reported usual sleep duration and their association on cognitive function (Teräs et al. 2020). The long sleepers tended to have worse cognitive function when compared to mid-range sleepers measured with either method of evaluating sleep duration. However, other previous studies using both methods to evaluate sleep duration have produced mixed results (McSorley et al. 2019; Scarlett et al. 2021). Both studies showed some associations accelerometer-measured short sleep duration and worse cognitive function, but one did not find associations between self-reported sleep duration and cognitive function (McSorley et al. 2019), while the other found long self-reported sleep duration to be associated with several cognitive domains (Scarlett et al. 2021).

There are some limitations to this study that need to be addressed. Although utilization of the three study cohorts is a significant strength, the distributions of age, socioeconomic status, and usual sleep duration were different between the cohorts which might have had some influence on the results. Furthermore, the sleep measures in the main analyses were based on self-reports which are only moderately correlated with objective measures, with self-reports providing some overestimation on average (Lauderdale et al. 2008; van den BERG et al. 2008). The overestimation may be an issue especially in the TSHeC study cohort, as the sleep duration was assessed with two separate questions of usual bedtime and wake time, which does not consider wakefulness after sleep onset. In our analyses we used sleep duration categorized into three categories instead of treating it as a continuous variable. This approach was selected as we know from previous literature that the association between sleep duration and cognitive function is U-shaped (Wei et al. 2022; Zhang et al. 2021). Additionally, this categorization enabled us to compare the results between self-reported and accelerometer-measured sleep duration. Furthermore, a threshold of 1 h was used to examine the difference between usual and previous night’s sleep duration and therefore we were not able to evaluate the association of smaller changes in sleep duration on cognitive function. Finally, there might be some residual confounders that were not adjusted for, such as work-related stress and anxiety which have been previously linked to sleep and cognitive function (Sindi et al. 2017; Tan et al. 2023). However, in our study only a small proportion of the participants were still in working life, while most were retired and information on previous work-related stress was not available.

In conclusion, we found that usual long sleep duration is associated with worse learning and memory, information processing, and reaction time among older adults. Additionally, we showed that sleeping more than usually the night prior to cognitive testing is associated with worse information processing but not with other cognitive domains. On the other hand, these results suggest that sleeping less than usually the night prior to cognitive testing is not associated with performance in cognitive testing. More research on the previous night’s sleep, preferable with device-based measurements, is needed to better understand the role of previous night’s sleep duration, as well as previous night’s sleep difficulties, on cognitive test performance.
